# EEG discrimination of perceptually similar tastes

**DOI:** 10.1002/jnr.24281

**Published:** 2018-08-06

**Authors:** Camilla Arndal Andersen, Marianne Leonard Kring, Rasmus Holm Andersen, Ole Næsbye Larsen, Troels Wesenberg Kjær, Ulla Kidmose, Stine Møller, Preben Kidmose

**Affiliations:** ^1^ Department of Engineering Aarhus University Aarhus Denmark; ^2^ Division of Technology and Innovation DuPont Nutrition & Health Brabrand Denmark; ^3^ Department of Food Science Aarhus University Aarslev Denmark; ^4^ Department of Biology University of Southern Denmark Odense Denmark; ^5^ Neurophysiological Center Zealand University Hospital Roskilde Denmark; ^6^ Department of Clinical Medicine University of Copenhagen Copenhagen Denmark

**Keywords:** gustatory evoked potentials, multivariate pattern analysis of EEG, quantitative EEG analysis, subliminal taste perception, sweetening agents

## Abstract

Perceptually similar stimuli, despite not being consciously distinguishable, may result in distinct cortical brain activations. Hypothesizing that perceptually similar tastes are discriminable by electroencephalography (EEG), we recorded 22 human participants’ response to equally intense sweet‐tasting stimuli: caloric sucrose, low‐caloric aspartame, and a low‐caloric mixture of aspartame and acesulfame K. Time‐resolved multivariate pattern analysis of the 128‐channel EEG was used to discriminate the taste responses at single‐trial level. Supplementing the EEG study, we also performed a behavioral study to assess the participants’ perceptual ability to discriminate the taste stimuli by a triangle test of all three taste pair combinations. The three taste stimuli were found to be perceptually similar or identical in the behavioral study, yet discriminable from 0.08 to 0.18 s by EEG analysis. Comparing the participants’ responses in the EEG and behavioral study, we found that brain responses to perceptually similar tastes are discriminable, and we also found evidence suggesting that perceptually identical tastes are discriminable by the brain. Moreover, discriminability of brain responses was related to individual participants’ perceptual ability to discriminate the tastes. We did not observe a relation between brain response discriminability and calorie content of the taste stimuli. Thus, besides demonstrating discriminability of perceptually similar and identical tastes with EEG, we also provide the first proof of a functional relation between brain response and perception of taste stimuli at individual level.

**Significance Statement**

During a meal the brain can respond to subliminal taste differences below the threshold of conscious perception. Using quantitative electroencephalography (qEEG) we discriminated brain responses to perceptually similar tastes and indicated that perceptually identical tastes are also discriminable. Furthermore, we show that participants, who more consistently discriminated a taste pair, also had brain responses that were more discriminable. The successful discrimination of perceptually similar tastes using EEG paves the way for future low‐invasive studies on subliminal taste processing with high temporal resolution.

## 1 INTRODUCTION

In our daily lives we constantly process new sensory input, of which little enters the scene of the conscious mind. Instead, it remains subliminal that is, the stimulus is below the threshold of detection, regardless of the attention level (Dehaene et al., 2006). Nevertheless, subliminal stimuli are known to be processed by the brain and to affect our behavior (Brázdil et al., 1998; Kopeikina et al., 2015; Meneguzzo et al., 2014; Pellegrino et al., 2017; Shevrin & Fritzler 1968). Subliminal *taste* responses in the human brain, however, remain relatively unexplored.

To our knowledge, only two studies have investigated subliminal taste responses in the human brain (Chambers et al., 2009; Frank et al., 2008). Both studies used fMRI and investigated subliminal taste responses by comparing taste stimuli with subliminal taste differences. The studies both investigated whether the taste of caloric and low‐caloric sweeteners showed distinct brain activations. Frank et al. (2008) found that brain responses to caloric sucrose and the low‐caloric sweetener, sucralose, were discriminable despite being perceptually identical. Likewise, Chambers et al. (2009) found that the perceptually identical caloric glucose and low‐caloric sodium saccharin were discriminable. Interestingly, Chambers et al. (2009) also replicated the finding from Carter et al. (2004), who showed that perceptually identical tastes can evoke distinct behavioral responses: rinsing the mouth with a caloric sweetener compared to a low‐caloric sweetener enhanced physical performance during a cycle time trial after fasting. Both Frank et al. (2008) and Chambers et al. (2009) suggested that brain responses were discriminated based on subliminal calorie detection in the oral cavity.

In fact, calorie detection has been supported by several studies (Spector & Schier, 2016). For example, two studies using mice proposed an additional receptor mechanism for caloric mono‐ and disaccharides, operating independently from the canonical T1R2 + T1R3 heterodimeric sweetness receptor (Sukumaran et al., 2016; Yee et al., 2011). If the T1R‐independent receptor mechanism also exists in humans, the implication is that caloric monosaccharides and disaccharides (e.g., glucose and sucrose) would be detected not only by the T1R2 + T1R3 receptor, as sweeteners are, but also via a separate T1R‐independent receptor mechanism. This could account for the supposed calorie detection by Chambers et al. (2009) and Frank et al. (2008).

The T1R‐independent detection could therefore account for the often disparate taste of non‐caloric sweeteners and sucrose, a popular caloric sugar (Larson‐Powers & Pangborn, 1978). Alternative explanations include different binding and affinity mechanisms to the T1R2 + T1R3 heterodimer‐receptor (Cui et al., 2006), binding to different taste quality receptors (Pronin et al., 2004), in addition to participant specific taste sensitivities affected by, for example, discrimination thresholds. These vary from participant to participant and from sweetener to sweetener (Peng et al., 2016).

So far, no EEG study has investigated subliminal taste differences. The reason is, no doubt, that tastes are harder to discriminate with EEG if they are hard to discriminate perceptually (Crouzet et al., 2015). EEG taste researchers have therefore ensured that their experimental designs elicited distinct taste percepts. Nevertheless, we hypothesized that perceptually similar and identical tastes are discriminable by EEG, and furthermore that discriminability is related to individual participants’ perceptual ability to discriminate the stimuli. EEG would, in contrast to fMRI, provide a highly detailed account of the temporal dynamics behind subliminal taste processing.

In the present study, taste stimuli were equi‐intense caloric sucrose, low‐caloric aspartame, and a low‐caloric mixture of aspartame and acesulfame K, which better mimics the taste of sucrose than aspartame (von Rymon Lipinski, 1985). The design of the experiment therefore allowed us to infer whether brain responses were mainly discriminated based on calorie detection or taste related differences. If calorie detection was a main discriminating factor, it would result in equally high discrimination of the brain responses to caloric sucrose and either of the low‐caloric stimuli, and low discrimination of the two low‐caloric stimuli. If calorie detection was not the main discriminating factor, then discrimination of the stimuli would be expected to reflect the participants’ perceptual ability to discriminate the taste stimuli.

## 2 METHOD

The method is divided in three main sections: (a) a *stimulus selection study* by a trained sensory panel; (b) an *EEG study* on untrained participants; (c) a *behavioral study* on the same untrained participants. The aim of the stimulus selection study (section 2.1) was to determine equi‐intense sweet taste stimuli for the EEG and behavioral study. The aim of the EEG study (section 2.2) was to record the brain responses to the taste stimuli, and the aim of the behavioral study (section 2.3) was to assess the participants’ perceptual ability to discriminate the taste stimuli.

### 2.1 Stimulus selection study

The aim of the stimulus selection study was to select sweet taste stimuli for the EEG and behavioral study that were equi‐intense in sweetness with 10% sucrose.

#### 2.1.1 Sensory equi‐sweetness test

A trained panel of eight assessors (six females and two males, mean age ± *SD*: 51 ± 10 years) at DuPont Nutrition & Health performed a sensory equi‐sweetness test according to the general guidelines for establishing a sensory profile (ISO‐13299, 2003) of four aqueous sweet stimuli: (a) sucrose at 100g/L (Dansukker, Nordic Sugar, Denmark); (b) aspartame at 0.45g/L (Ajinomoto Sweeteners, France); (c) acesulfame K at 0.34g/L (Nutrinova, Germany); (d) 60–40% mixture of aspartame and acesulfame K at 0.23g/L (0.14g/L of aspartame and 0.09g/L of acesulfame K). The concentration level was based on an initial taste screening, and all stimuli were additionally prepared in two higher and two lower concentrations such that the concentration of successive stimuli was 1.5 times that of the previous.

The assessors were tested, selected and trained according to the general guidelines for the selection, training and monitoring of selected assessors and expert sensory assessors (ISO‐8586, 2012). Samples were evaluated in the whole oral cavity on the attributes: (a) maximum sweetness; (b) sweet duration; (c) time before sweet taste; (d) metallic; (e) artificial‐chemical; (f) bitter; (g) viscosity. All attributes were evaluated on an unstructured scale (0–100) with anchors at 10 and 90 which the assessors were trained to correspond to minimum sweet intensity (non‐chlorinated tap water) and maximum sweet intensity (sucrose 225g/L), respectively. Twenty taste samples (four taste stimuli on five concentration levels) of 30 mL were blind‐labeled with seemingly random numbers to conceal the stimulus identity and served one at a time in balanced‐randomized order in four replicates. The assessors evaluated all attributes for each sample. The samples were served at ambient temperature, around 21°C, and could not be discriminated via the visual or olfactory system since all samples looked like water and had no odor. Samples were served in 50 mL cups with lids (PS‐978, Emballator Växjöplast, Sweden). Non‐chlorinated tap water and crisp bread was available during testing to neutralize taste sensation between samples and limit carry‐over effects.

#### 2.1.2 Stimulus selection analysis

The pure acesulfame K solution was discarded as a stimulus for the EEG and behavioral studies since it produced very strong off‐flavors at high concentrations. The concentration of the taste stimuli was determined by interpolating their maximum sweetness scores to that of 10% sucrose. The interpolation was based on the average response across participants modeled by quadratic spline fits in piecewise polynomial form smoothed to optimize the fit (MATLAB 2017a, MathWorks, Natick, Massachusetts, United States). Based on the interpolation, the stimuli for the EEG experiment were chosen to be: (a) aqueous sucrose (100g/L) referred to as ‘Suc’; (b) aqueous aspartame (0.94g/L) referred to as ‘Asp’; (3) aqueous mixture of aspartame and acesulfame K (60% aspartame, 0.228g/L, and 40% acesulfame K, 0.158g/L) referred to as ‘Mix’.

#### 2.1.3 Taste stimuli differences

The taste stimuli, Suc, Asp, and Mix were determined by aligning their scores of the maximum sweetness attribute. Enabled by the five additional attributes that were also evaluated in the stimulus selection study (sweet duration, time before sweet taste, metallic, artificial‐chemical, bitter, and viscosity), we estimated potential differences of Suc, Asp, and Mix in the EEG and behavioral studies. For each attribute and assessor, the score of Asp and Mix stimuli was estimated by interpolating the responses in the sensory profile. To avoid underestimating variability of the interpolated scores, the variance was adjusted to the mean variance from the two nearest concentrations. Differences between the Suc, Asp, and Mix were evaluated by ANOVA on each attribute with participant as a random factor. The model was: A_ti_ = µ + α_t_ + S_i_ + ɛ_ti_, where A = attribute score, t = taste (Suc, Asp, Mix), i = individual subject number and ɛ_ti_ = random residual (R, The R Foundation, version 3.3.1). Correction for multiple comparisons was performed using Fisher's least significant difference.

### 2.2 EEG study

#### 2.2.1 Participants

Twenty‐four volunteers participated in the study without remuneration after giving an oral and written informed consent (16 females and 8 males, mean age ± *SD*: 34 ± 8 years). The protocol was approved by The Central Denmark Region Committees on Health Research Ethics (reference number: 1–10‐72–294‐16) and was conducted according to the Declaration of Helsinki. The number of participants was chosen to be in the same range as recent comparable studies (Crouzet et al., 2015; Jacquin‐Piques et al., 2015, 2016; Tzieropoulos et al., 2013). Only volunteers with self‐reported normal taste perception were accepted. Volunteers outside the age range 18–50 years and those who smoked were excluded.

#### 2.2.2 Experimental protocol

Participants were recorded at 9 a.m. They were instructed to refrain from menthol, spicy food, and coffee on the day of recording, and were only allowed to consume water an hour up to the EEG recording. During recording, the participants were instructed to position the head in a chin rest and protrude the tongue out of the mouth. Taste stimuli were administered to the center of the tongue's apex by programable pumps (NE‐1010, World Precision Instruments, USA) through one common nozzle to ensure identical stimulation site on the tongue for all stimuli. Each taste stimulation was cued 3 s before onset via a computer screen, and during stimulus presentation the participant was instructed to focus on a cross on the computer screen, not to move, and not to blink. The taste stimuli flowed off the tongue and were collected in a beaker to avoid swallowing behavior. After each stimulation the tongue was rinsed with 9 mL non‐chlorinated water. All stimuli were adjusted to 21°C to eliminate temperature differences. To eliminate confounding auditory evoked potentials, the pumps were kept in a sound attenuating box and music of the participant's preference was played through in‐ear headphones.

Stimulus duration was three seconds, and stimulus volume was 5.25 mL. Each taste stimulus was repeated 60 times. However, four participants produced many artifacts throughout the recording and were therefore served up to 72 repetitions to ensure adequate data for analysis. On average, the number of stimulus repetitions was therefore 62 across all participants. The order of taste stimulation was randomized so neither the participant nor the lab technician knew the sequence. All three taste stimuli were administered on the tongue without preceding tactile stimulation, thereby simultaneously evoking both a somatosensory response (tactile and temperature sensations) and a taste response. However, since the three taste stimuli evoked identical tactile and temperature sensations any difference between the taste stimuli could be assumed to reflect taste.

#### 2.2.3 EEG recording setup

Brain activity was measured with 128‐channel EEG (ANT Neuro, Netherlands) with channels positioned according to the 10–20 system. Channel impedances were maximally 10 kOhm. A ground channel was placed on the left wrist, and eye movement was estimated with horizontal electrooculogram. Data was sampled at 512 Hz and passed through the built‐in analog low‐pass filter with a cutoff of 138 Hz.

#### 2.2.4 Preprocessing of EEG data

Data was bandpass filtered from 1 to 30 Hz (EEGlab version 14.1.1b, Hamming windowed sinc FIR filter, 1690 filter coefficients) (Delorme and Makeig 2004). The timing of stimulus onset was adjusted based on sensors at the tip of the nozzle that detected stimulus arrival by a decrease in electrical resistance between the sensors. Data was extracted −0.2 to 1.0 s relative to stimulus onset, and each trial was baseline corrected relative to the 0.2 s prestimulus period. All 128 scalp channels were re‐referenced to average reference, defined as the average of all but the rejected channels.

Trial‐channel pairs were identified as noisy based on an amplitude criterion (absolute amplitude larger than 60 µV) and a trend criterion (> 50 µV slope per trial if R^2^ > 0.3 for a linear fit). If 1–2 channels in a trial met a rejection criterion, they were interpolated from the remaining channels in that trial (spherical interpolation, EEGlab version 14.1.1b). If three or more channels in a trial met a rejection criterion, then the entire trial was marked for rejection. Channels were rejected in all trials if they caused more than 10% of the trials to be marked for rejection for either one of the three stimuli for the same participant. Rejected channels were then interpolated from the remaining channels. The set of trials marked for rejection was revised upon manual inspection, and the marked trials were then removed. On average 60 trials were accepted for all conditions for every participant. All 24 participants were accepted for further analysis.

For each taste condition, grand‐average evoked potentials were estimated by averaging across all accepted trials for each participant (referred to as *within‐participant averaged evoked potentials*), and then averaging across participants.

#### 2.2.5 Global field power

We used global field power to illustrate the temporal dynamics of taste responses (Skrandies, 1990). Global field power shows the global activity across the scalp. It was computed for all taste conditions as the standard deviation across channels at every time sample for every participant, and then averaged across participants.

#### 2.2.6 Cluster permutation test

We used cluster permutation test to test significance of the brain responses to the taste stimuli (the grand‐average evoked potentials) and to test for differences between each pair of the three taste responses. The cluster permutation test is state of the art within EEG analysis. It extends the classic permutation test by incorporating a proximity constraint, which prioritizes effects that are close in time and space, such as brain processes, and unlike noise components (Maris & Oostenveld, 2007). The cluster permutation test requires a threshold to base the clustering on. The threshold does not affect the test's validity (Maris & Oostenveld, 2007), and in the present paper, threshold values were chosen to keep computing‐time within a practical time frame. The advantage of the cluster permutation test is that it solves the multiple comparisons problem, and furthermore that its validity, as a nonparametric test, does not depend on the input data's probability distribution.

In the present study, the cluster permutation test was implemented to test significance of the grand‐average evoked potentials by performing a two‐tailed *t* test to estimate difference from zero of the within‐participant averaged evoked potentials at every sample for all channels. Then *t*‐values above a threshold of *t* = 4 were clustered across time (adjoining samples) and space (channels within a radius of 30 mm). The procedure was repeated 2,000 times on data with time points permuted on participant level. The largest cluster in each repetition, evaluated by the sum of *t*‐values in the cluster, served as a permutation distribution. Statistical significance (*p*‐values) of each of the clusters from the original, unpermuted data set was then estimated as the proportion of clusters in the permutation distribution that were the same size or larger. To test for differences between each pair of the three grand‐average evoked potentials, the cluster permutation test was implemented in a slightly different way: for each taste pair, a paired *t* test was performed at every sample for all channels to estimate the difference between the within‐participant averaged evoked potentials. *T*‐values above a threshold of *t* = 3 were then clustered across time and space, and the permutation procedure repeated on data with taste labels shuffled on participant level.

#### 2.2.7 Quantitative EEG analysis method

In order to exploit the multivariate nature of high density EEG, a quantitative EEG analysis method (qEEG) was applied to discriminate taste responses. The qEEG was implemented in a slightly modified form of the time‐resolved multivariate pattern analysis from Crouzet et al. (2015). Discrimination was performed with a logistic regression classifier trained to discriminate the taste responses at single‐trial level, thus at every time sample for all trials and participants, yielding classification with a temporal resolution of 512 Hz (Pattern Recognition and Machine Learning Toolbox, ver. 1.0, Mathworks File Exchange). Data was normalized to a 0–1 range prior to training and testing, and the classifier was L2 regularized (λ = 1). To boost robustness to noise and trial‐to‐trial jitter, training included scalp map data from the surrounding 0.1 s of the test sample (0.05 s on each side), which were treated as independent and identically distributed scalp maps in the training phase, effectively increasing the amount of training data. Only the center test sample in the interval was used to evaluate each classifier. The training and evaluation was repeated for all time samples and averaged across trials and participants. A slight variation of the analysis was implemented on within‐participant averaged evoked potentials, as an alternative to the single‐trial analysis.

Two decoding approaches were used: between‐participant and within‐participant. Between‐participant decoding assumed that participants shared neural response patterns. Training and testing were performed by leave‐one‐participant‐out cross validation. For every iteration, data from one participant was excluded from the training data, a model trained on the remaining participants, and tested on the excluded participant. This was repeated until all participants had been excluded once. Within‐participant decoding assumed that between‐participant variances were high compared to taste response differences and that a participant specific classifier was needed to reveal the subtle taste response patterns. Here, training and testing were performed by 10‐fold cross validation without replacement. The trials were divided into 10 subsets and at every iteration a model was trained on data from 9 subsets and tested on the remaining subset until all subsets had been tested once. The procedure was repeated for all participants. The output of qEEG from each sample‐trial pair was a decoding probability for each taste category. The decoding probability for correct classification was used as a measure of qEEG's certainty of the classification and its significance above chance level was estimated by a right tailed *t* test at every sample. To adjust for multiple comparisons, the effect was only considered significant if *p* ≤ 5% for a continuous time interval of at least 100 ms, as in the comparable study by Crouzet et al. (2015).

### 2.3 Behavioral study

The participants in the EEG study were subsequently asked to perform a behavioral discrimination task on a separate occasion. The separation of the EEG and behavioral studies served to limit taste adaptation effects, and to ensure that behavioral effects, such as contingent negative variations, would not be present in the EEG data. Two participants were unable to attend the discrimination task and were therefore excluded from the aggregate EEG data set, reducing the number of participants to 22 (14 females and 8 males). The discrimination task was performed as a triangle test, where participants had to identify which of three taste samples was different from the other two. The discrimination task was performed for all three taste pairs, that is, Suc versus Asp (SA), Suc versus Mix (SM), and Asp versus Mix (AM). Each taste pair was repeated six times, once for each of the six possible serving sequences to avoid carry‐over effects in the final result. The total of 18 repetitions (six repetitions of three taste pairs) were randomized. The taste stimulation setup was the same as in the EEG study, but without the EEG cap. Consequently, only the center of the tongue's apex was stimulated and the sensory input was therefore drastically reduced compared to the stimulus selection study (section 2.1). To discriminate a taste pair at 10% significance level the participants had to discriminate four of the six repetitions, and five of the six repetitions to discriminate the stimuli at 5% significance level (binomial distribution, without adjusting for multiple comparisons, MATLAB 2017a). Whether sex affected the ability to discriminate the taste pairs was estimated by Fisher's exact test at 5% significance level (MATLAB 2017a). The test was based on the ratio of male and female participants who were able to discriminate at least one taste pair at a 10% significance level against the participants who were not able to discriminate any taste pair.

## 3 RESULTS

With the aim to study subliminal taste responses, we optimized three taste stimuli to produce similar taste percepts. In the EEG study we then recorded the taste responses on 22 participants, and in the behavioral study we assessed the participants’ perceptual ability to discriminate the tastes. By careful design of stimuli and setups, we ensured that the EEG and behavioral studies only allowed the stimuli to be discriminated based on the sense of taste.

### 3.1 Behavioral study

To verify that the tastes were perceptually similar or identical, we assessed the participants’ perceptual ability to discriminate each of the taste pairs.

Table 1 shows the participants’ perceptual ability to discriminate the three taste pairs: SA, SM, and AM. For each participant, the number of correct identifications of six repetitions is reported. Significant identification at 10% significance level is highlighted with bold font, and at 5% significance level with gray background.

**Table 1:** Participants' perceptual ability to discriminate each of the three taste pairs: Suc versus Asp (SA), Suc versus Mix (SM), and Asp versus Mix (AM)

Participant	Sex	SA	SM	AM	Mean
1	F	2	2	1	1.7
2	F	1	4*	2	2.3
3	F	4*	2	3	3.0
4	F	3	2	1	2.0
5	F	2	2	3	2.3
6	F	1	1	2	1.3
7	F	4*	2	0	2.0
8	M	1	2	2	1.7
9	F	2	3	2	2.3
10	M	5**	1	4*	3.3
11	M	5**	4*	3	4.0
12	M	2	2	2	2.0
13	F	4*	2	2	2.7
14	F	1	2	1	1.3
15	F	4*	4*	3	3.6
16	M	1	0	2	1.0
17	F	5**	2	3	3.3
18	F	2	2	3	2.3
19	M	4*	4*	4*	4.0
20	M	1	1	5**	2.3
21	F	5**	3	2	3.3
22	M	3	4*	1	2.7
**Mean**		2.8	2.3	2.3	

The number of correct identifications of 6 repetitions for each taste pair is shown for each of the 22 participants. Discrimination at a 10% significance level is indicated with one asterisk and at a 5% significance level with two asterisks (binomial distribution). The participants' sex is shown in the first row; female (F) and male (M).

None of the participants were able to discriminate all six repetitions correctly for any taste pair. Only in 5 of the 66 discrimination tests (corresponding to 8%) did the participants significantly discriminate the taste pairs: no participant significantly discriminated SM, one participant discriminated AM, while four participants discriminated SA (α = 5%, Table 1). Thus, of the three taste pairs, SA was discriminated best, yet, on average, the participants identified less than half of the six SA repetitions correctly (2.8 of 6, Table 1). Considering the influence of sex: at least one of the three taste pairs was discriminated at a 10% significance level for five of the eight participating men, and for seven of the fourteen women, (Table 1). The difference between sexes was not significant according to Fisher's exact test (*p* = .67).

The behavioral result strongly indicated that Suc, Asp, and Mix tasted similarly or even identical depending on the specific participant and taste pair. We chose to define that tastes were perceptually similar if the participant discriminated the taste pair at a 10% significance level (4–5 correct of 6), while tastes with poorer discrimination were defined as perceptually identical (0–3 correct of 6). Since the setup in the behavioral study matched the setup in the EEG study, this allowed us to assume that participants discriminated tastes comparably in the two setups. Thus, percepts of taste stimuli were either perceptually similar or identical in both the behavioral and EEG studies.

### 3.2 EEG study: Decoding based on within‐participant averaged evoked potentials

To test whether brain responses to Suc, Asp, and Mix were recorded, we assessed significance of their grand‐average evoked potentials. In addition, we also tested whether Suc, Asp, and Mix gave *different* brain responses by assessing whether their grand‐average evoked potentials could be discriminated. All analyses were performed at the level of within‐participant averaged evoked potentials (single trials were averaged within each taste condition for every participant).

**Figure 1:**
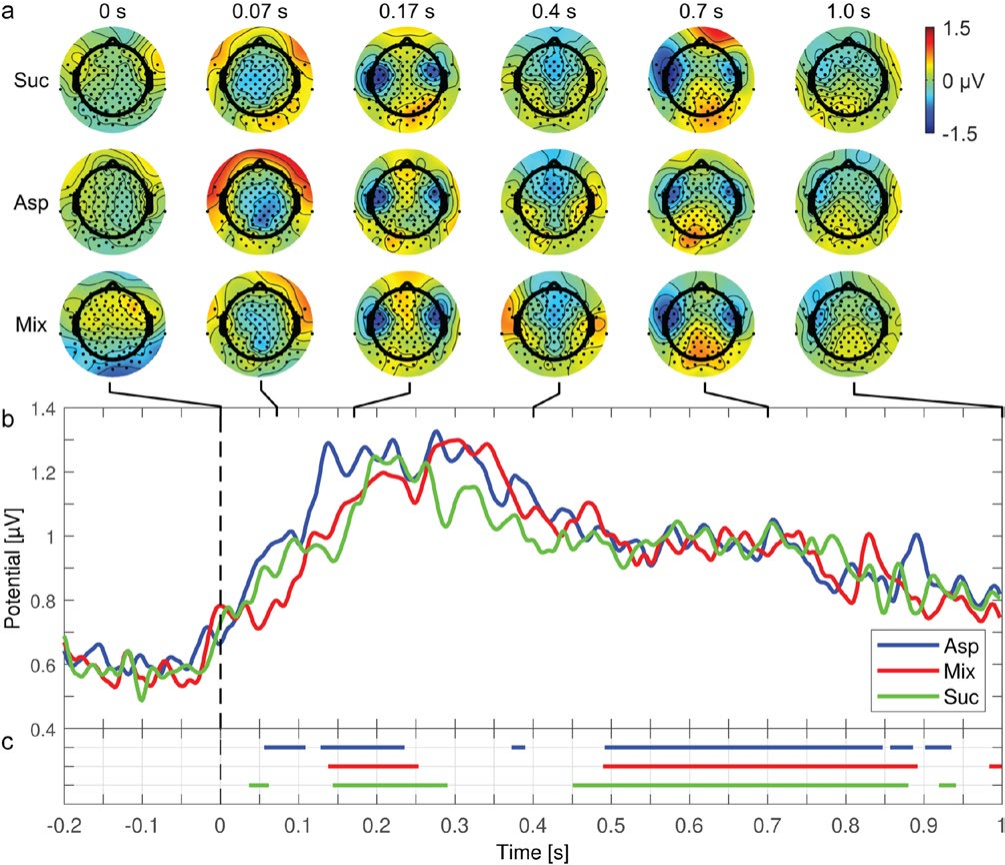
Grand‐average brain responses to the three taste stimuli: sucrose (Suc), aspartame (Asp), and a mixture of aspartame and acesulfame K (Mix) (a) Grand‐average scalp maps for Suc, Asp, and Mix. Color‐coding is relative to the absolute maximum potential difference. The position of all 128 channels is marked with black dots. (b) Global field power for Suc, Asp, and Mix. (c) Time periods where the brain responses to Suc, Asp, and Mix were significantly different from zero according to a cluster permutation test on within‐participant averaged evoked potentials. The cluster permutation test did not reveal differences between the within‐participant averaged evoked potentials and the result is therefore not shown [Color figure can be viewed at wileyonlinelibrary.com]

Figure 1 shows the average brain response for each stimulus: Suc, Asp, and Mix. Figure 1a illustrates grand‐average scalp plots at 0.0, 0.07, 0.2, 0.4, 0.7, and 1.0 s post stimulus. The time instances were chosen to illustrate a control period (0.0 s), as well as clear activations and long‐term trends. Channels are marked with black dots. Figure 1b shows the temporal development of the evoked potentials by their global field power averaged across participants. Figure 1c illustrates when the grand‐average evoked potentials were significantly different from zero (cluster permutation test, threshold = 4).

All three brain responses followed the same overall pattern in both the spatial (Figure 1a) and temporal domain (Figure 1b) and indicated high reproducibility of the responses. As expected, the evoked potentials had arbitrary topographical patterns at 0.0 s. At 0.07 s there was a central negative deflection that was replaced by negative deflections at both fronto‐temporal lobes after 0.2 s. At 0.4 s the negative deflection moved to a fronto‐central area, but then returned to the fronto‐temporal lobes at 0.7 s where it grew weaker as seen after 1.0 s. In the temporal domain the activity of all three taste responses increased after stimulus onset, plateaued from 0.15 to 0.35 s, and then declined again as indicated by global field power (Figure 1b).

All three grand‐average evoked potentials were significant according to a cluster permutation test on the within‐participant averaged evoked potentials (Figure 1c). Clusters were found in the period from 0.04 to 1.0 s. However, the cluster permutation test did not find significant differences between the grand‐average evoked potentials (result not shown), confirming that the brain responses to Asp, Mix, and Suc followed the same overall pattern. An additional attempt was made to discriminate the responses using qEEG on the within‐participant averaged evoked potentials, but without success: continuous significant decoding was maximally 12 ms (from 0.136 to 0.148 s, result not shown).

Thus, significant brain responses to the taste stimuli were recorded, but could not be discriminated based on their within‐participant averaged evoked potentials.

### 3.3 EEG study: Decoding based on single‐trial evoked potentials

Since tastes could not be discriminated based on within‐participant averaged evoked potentials, our ambition was to discriminate the tastes based on evoked potentials at single‐trial level. Discrimination was performed with qEEG trained and implemented using two separate approaches: either on patterns between‐participants or on patterns within‐participants.

**Figure 2:**
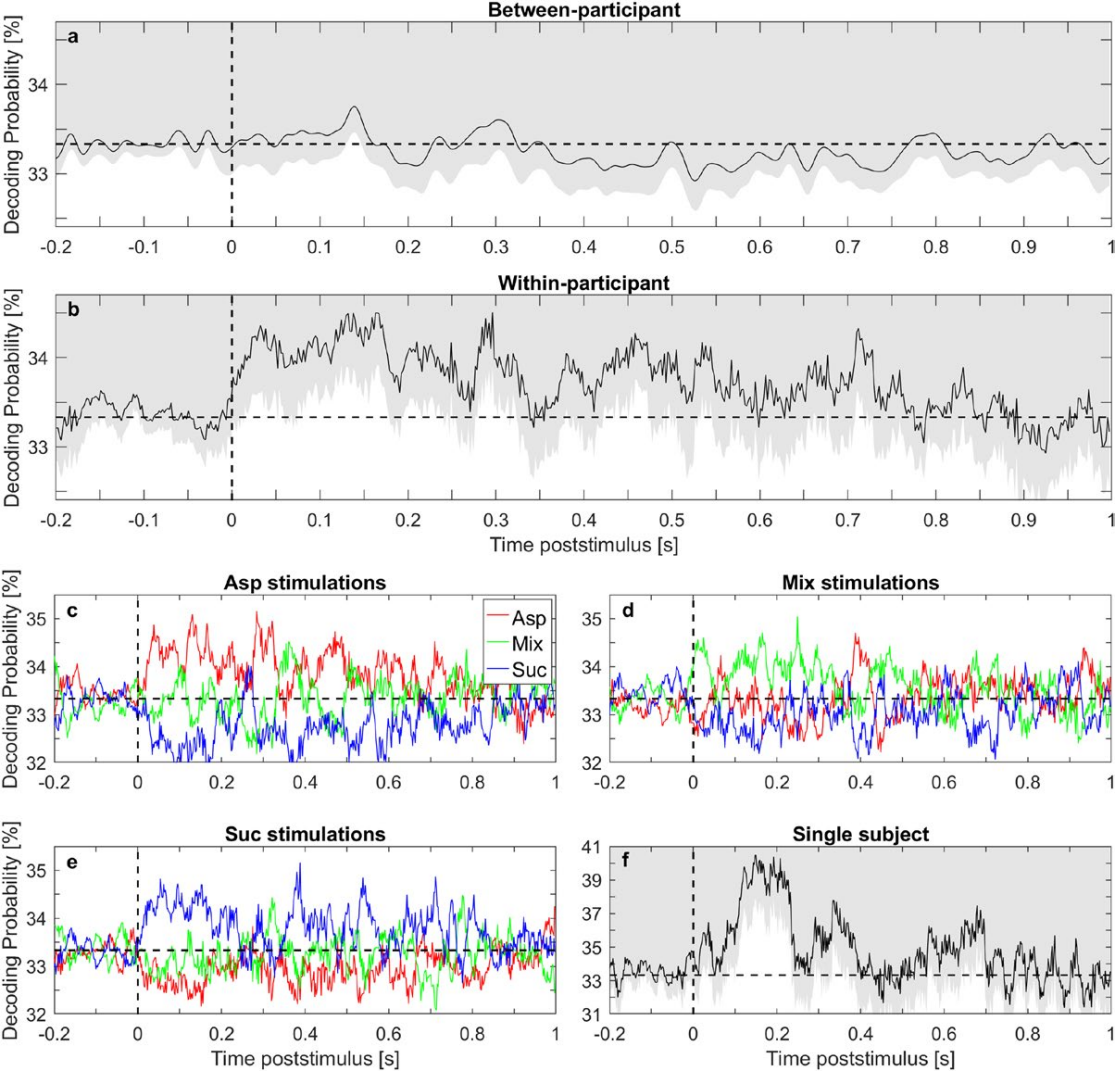
Multi‐class decoding based on evoked potentials to sucrose (Suc), aspartame (Asp), and a mix of aspartame and acesulfame K (Mix) using quantitative EEG analysis (qEEG). For each participant, a logistic regression classifier was trained to discriminate Asp, Mix, and Suc based on data from the participant itself (within‐participant) or from the remainder of the participants (between‐participant). Average decoding probability across taste stimuli and participants is illustrated with a right tailed 95% confidence interval for (a) between‐participant and (b) within‐participant analysis. The underlying classification of each of the taste stimuli is detailed for the within‐participant analysis: (c) Asp, (d) Mix, and (e) Suc. (f) An example of a single participant decoding based on within‐participant qEEG, as opposed to the average participant decoding in (b). Horizontal lines at 33⅓% illustrate chance level [Color figure can be viewed at wileyonlinelibrary.com]

Figure 2 illustrates decoding probability by qEEG based on three‐class logistic regression classifiers with classes: Asp, Mix, and Suc (chance level 33⅓%). For every participant, qEEG was trained on either the remainder of the participant group (between‐participant), or on the participant itself using 10 cross‐validation steps (within‐participant). Decoding probability was then averaged across participants. Average decoding probability across taste stimuli is illustrated with a right tailed 95% confidence interval for between‐participant analysis (Figure 2a) and within‐participant analysis (Figure 2b). The underlying within‐participant decoding of Asp is plotted in Figure 2c, of Mix in Figure 2d, and of Suc in Figure 2e. An example of within‐participant multi‐class decoding of a *single* participant is shown in Figure 2f, as opposed to the participant mean in Figure 2b.

Taste response discrimination by between‐participant qEEG (Figure 2a) was clearly inferior to discrimination by within‐participant qEEG (Figure 2b,f). Within‐participant qEEG decoding probability for all three taste stimuli increased from chance level at 33⅓% after stimulus onset and returned to chance level again after approximately 0.75 s (Figures 2b–e). The longest interval with significant discrimination of the three taste stimuli was 0.1 s from 0.08 to 0.18 s (Figure 2b). In a comparable study by Crouzet et al. (2015) multiple comparisons were arbitrarily adjusted “using a time‐cluster approach in which a time point was considered significant only when it was a member of a cluster of at least four consecutively significant time points,” corresponding to 0.1 s in their study. Using this convention, the period from 0.08 to 0.18 s was significantly above chance level, thus the taste responses were significantly discriminated (Figure 2b). Particularly, Asp and Suc were discriminated from the other taste stimuli, and especially from each other (Figure 2c,e). In contrast, the responses to Mix were more often misclassified and confused with Asp and Suc (Figure 2d).

Summing up, within‐participant qEEG on single‐trial level successfully discriminated brain responses to the three taste stimuli (Figure 2b). Coupled with the failed attempt of between‐participant qEEG to discriminate the taste responses (Figure 2a), this indicates that between‐participant variances are high compared to taste response differences. Hence, recorded brain responses did not generalize across participants.

### 3.4 Relation between EEG and behavior on taste pair level

**Figure 3:**
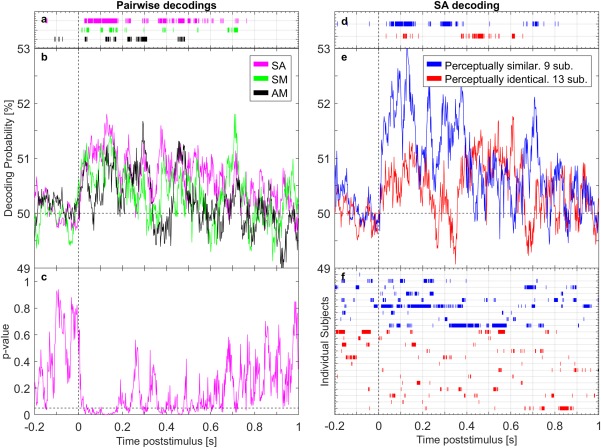
Two‐class decoding based on evoked potentials to sucrose (Suc), aspartame (Asp), and a mix of aspartame and acesulfame K (Mix) using within‐participant quantitative EEG analysis (qEEG). A classifier was trained to discriminate taste pairs: Suc and Asp (SA), Suc and Mix (SM), and Asp and Mix (AM). The left side of the figure shows qEEG decoding results from all three taste pairs. (a) Periods where the taste pairs were decoded significantly above chance are indicated by bars, and (b) decoding probability for each taste pair averaged across participants. (c) Statistical significance of the SA taste pair detailed at every time point (right tailed *t* test, α = 5%). The right side of the figure shows qEEG decoding results of the SA taste pair according to the participants’ ability to perceptually discriminate SA. qEEG was performed on perceptually similar taste responses (significant discrimination at 10% significance level, 9 participants) and on perceptually identical taste responses (13 participants). (e) Average decoding probability across participants for the perceptually similar and perceptually identical taste responses, with periods of significant decoding illustrated by bars on (f) an individual level (d) and subgroup level (right tailed *t* test, α = 5%). Horizontal lines at 50% illustrate chance level [Color figure can be viewed at wileyonlinelibrary.com]

Prompted by the successful discrimination of the taste pairs, we then assessed whether the discriminatory performance of qEEG could be linked to the participants’ perceptual ability to discriminate the taste stimuli. Within‐participant qEEG at the single‐trial level was therefore performed on each taste pair (Figure 3a–c) to allow direct comparison to the participants’ performance in the behavioral study (Table 1). We then assessed whether there was a relation between EEG and behavior at *taste pair* level, such that taste pairs that were perceptually hard to discriminate, were also harder to discriminate by qEEG. The result would also indicate whether calories were a main discriminating factor since this would lead to poor discrimination of the two non‐caloric sweeteners (AM), and higher discrimination of the taste pairs with caloric sucrose and non‐caloric sweetener (SA and SM).

Figure 3a–c illustrates decoding by qEEG based on two‐class logistic regression classifiers with the taste pairs: SA, SM, and AM (chance level 50%). For every participant, qEEG was trained on the participant itself using 10 cross‐validation steps, and decoding probability was then averaged across participants. Figure 3a illustrates when the taste pairs were significantly decoded (right tailed *t* test, α = 5%), and Figure 3b shows the respective decoding. Significance of the SA taste pair decoding is detailed in Figure 3c.

Overall, decoding probability for all three taste pairs increased from chance level at 50% after stimulus onset and returned to chance level again after approximately 0.75 s (Figure 3b). The SA taste pair was generally best decoded and was, save for a few time samples, significantly decoded in the period from 0.03 to 0.18 s (Figure 3a). Decoding of the SM and AM taste pairs followed the same general pattern as the SA decoding, although with a lower decoding probability, especially for AM taste pair (Figure 3b).

The SA taste pair was discriminated best both perceptually and by qEEG, indicating a relation between EEG and behavior on a *taste pair* level. The result therefore replicates the conclusion by Crouzet et al. (2015): average discriminability of a taste pair by qEEG and by perception are related, now shown for taste differences at or below discrimination threshold.

We saw no evidence of a calorie detection as this would have led to equally high discrimination of the SA and SM taste pairs. Instead, decoding probability of the taste pairs reflected the perceptual ease at which they were discriminated.

### 3.5 Relation between EEG and behavior on the individual level

The participants could be split into two subgroups of roughly the same size based on their perceptual ability to discriminate the SA taste pair: participants who found Suc and Asp to be similar and participants who found them to be identical. This enabled us to analyze whether qEEG can decode perceptually similar and perceptually identical tastes. In extension thereof we also assessed whether there was a relation between EEG and behavior at *individual* level, such that participants who were better at discriminating the SA taste pair perceptually also had brain responses showing the same discrimination.

Figure 3d–f illustrates the relation between EEG and behavior at the individual level for the SA taste pair. Decoding was performed by qEEG as a within‐participant analysis at single‐trial level on perceptually similar taste responses (significant discrimination at 10% significance level, 9 participants) and perceptually identical taste responses (13 participants). Decoding probability of both the perceptually similar and the identical taste responses are plotted in Figure 3e. Their significance from chance level at 50% (right tailed *t* test, α = 5%) is plotted at subgroup level in Figure 3d and at participant level in Figure 3f. The SM and AM taste pairs only had few perceptually similar responses (3–5 participants), and were therefore not analyzed.

qEEG's discrimination of both perceptually similar and identical tastes increased from 0.05 to 0.17 s, but with superior decoding for perceptually similar tastes (Figure 3d,e). As seen in Figure 3f, the difference was caused by a general trend among the participants, with no single participant driving the group mean.

The analysis revealed that qEEG can discriminate perceptually similar taste responses, and (to a certain extent) perceptually identical taste responses (Figure 3d,e). The analysis also revealed that brain responses are better discriminated when tastes are perceptually similar than when identical. The tendency was also seen among individual participants (Figure 3f), and for the first time provides evidence of a link between perception and brain response on an individual level. In general, participants who more consistently discriminated a taste pair perceptually also had better discriminated brain responses.

Interestingly, the decoding of perceptually similar and perceptually identical tastes resembled each other: the prestimulus period was at chance level, and shortly thereafter qEEG classification improved for a period of about 0.2 s (Figure 3e). It therefore seems highly probable that the tastes were discriminated by the brain, but that they were too similar to be perceptually discriminable. To our knowledge, this is the first time that decoding of perceptually identical tastes has been indicated with EEG.

## 4 DISCUSSION

Based on the stimulus selection study, we chose three stimuli with sweet taste of equal intensity. We found that the three taste stimuli were perceptually similar or identical in the behavioral study, yet significantly discriminable from 0.08 to 0.18 s by qEEG. Brain response discrimination was achieved by qEEG trained on patterns within subjects, instead of generalizing across participants. This suggests that variances in the participants’ brain responses were high compared to taste response differences. Comparing the participants’ responses in the EEG and behavioral studies, we can summarize the main results of the present investigation as follows: (a) Brain responses to perceptually similar tastes and (to a certain extent) perceptually identical tastes can be discriminated; (b) Discriminability was related to participants’ perceptual ability to discriminate the tastes, both at taste pair and individual level. Thus, for the first time we provide evidence for discrimination of perceptually similar and identical tastes with EEG and relate discrimination to individual perception.

As opposed to earlier studies on taste evoked potentials (Franken et al., 2010; Hummel et al., 2010; Iannilli et al., 2014, Jacquin‐Piques et al., 2016), the taste stimuli in the present study were optimized to produce similar taste sensations. Perceptual similarity of the taste stimuli was assessed for each participant in the behavioral study and assumed to apply to their sensations in the EEG study. However, since the EEG study, unlike the behavioral study, did not enforce discrimination tasks and hence attention on taste differences, the taste percepts in the EEG study were at most as distinguishable as in the behavioral study. The similarity of the taste evoked potentials in the EEG study could therefore be explained by the similar taste percepts and consequently similar brain processes. However, by applying a quantitative EEG analysis (qEEG), we were able to discriminate perceptually *similar* tastes and furthermore able to indicate discrimination of perceptually *identical* tastes. This suggests that taste differences can be subconsciously discriminated by the brain, even though they are too similar to be discriminated perceptually. The result is in accordance with the fMRI studies by Frank et al. (2008) and Chambers et al. (2009), who found that differential brain responses can be elicited by subliminal taste differences. They suggested that brain responses were discriminated based on subliminal calorie detection. We, however, have not been able to confirm calorie detection. Had calories been a main discriminable factor, then the SM taste pair should have been as discriminable as the SA pair.

Compared to fMRI, EEG allowed us to investigate subliminal taste processes with higher temporal resolution. Our results revealed that discrimination mainly occurred from 0.08 to 0.18 s for both perceptually similar and perceptually identical tastes. Previous EEG studies have also investigated taste discrimination, albeit with clearly distinct taste percepts. They found taste discrimination *onset* at 77 ms comparing two salty concentrations (Tzieropoulos et al., 2013), at 370 ms comparing salty, umami, sweet, acid, and bitter stimuli (Iannilli et al., 2017), at 400 ms comparing a sweet and a taste neutral stimulus (Franken et al., 2010), and at 175 ms comparing salty, sweet, sour, and umami stimuli (Crouzet et al., 2015). Thus, previous studies have found taste discrimination onsets in a wide spectrum ranging from 77 to 400 ms. This may be due to variations in taste stimuli and experimental setups, besides the studies’ fundamentally different analysis methods. The present study is, however, closely related to the study by Crouzet et al. (2015), which observed taste discrimination onset approximately 100 ms later than in the present study. The delay may partly be explained algorithmically, since qEEG in the present study was allowed to train on data 0.05 s before and after each classified time point; thus, enabling early stages of brain patterns to be recognized by training on clearer late stage patterns. Alternatively, it could also be explained physiologically, since taste onset in the present study was paralleled by a somatosensory onset, much like in everyday eating situations. Somatosensation could therefore alter the timing of brain responses via interactions between the gustatory and somatosensory system.

Regardless of the cause behind the deviating taste discrimination onsets, it is clear, that brain responses in the present study were discriminated at a comparably early time point at 80 ms, coinciding with the earliest onset of taste discrimination in the related body of literature (Tzieropoulos et al., 2013). This suggests that brain response discrimination was based on primary sensory taste processes, and not later cognitive brain processes.

Whereas the stimuli had equal sweet intensity, they deviated on other taste attributes. This could account for brain response discriminability in the primary sensory system. According to the stimulus selection study, Suc and Asp were significantly different on all attributes except maximum sweetness, Suc and Mix were significantly different on four of the attributes (artificial sweet, metallic taste, bitterness, and thickness), while Asp and Mix were not significantly different on any attribute. Based on the stimulus selection study we therefore propose that taste stimuli in the EEG study were not only discriminated based on sweetness related differences, but also on other taste qualities such as bitterness. Yet, this must only be seen as a cautious proposal due to the disparate setups in the stimulus selection study and EEG study: the stimulus selection study was performed on trained assessors who were given 30 mL of sample material and were allowed to taste the stimuli in the entire oral cavity, while the participants in the EEG study were untrained and only served 1 mL of the taste stimuli onto the tip of the tongue.

Crouzet et al. (2015) was the first study to relate discriminability of tastes between EEG and behavioral measures. The study operated with distinct tastes and performed analysis at *taste pair* level: they averaged discriminability of EEG and behavioral responses across participants before comparing taste pairs. The study found a relation between EEG and behavior: taste pairs, which on average were easy to discriminate perceptually, were also, on average, easy to discriminate based on their brain responses. We have replicated the result, now with taste differences at or below discrimination threshold: the SA taste pair was, on average, better discriminated than the SM and AM taste pairs in both the EEG and behavior study. The relation between EEG and behavior even exists across the two studies as the taste pairs in Crouzet et al. (2015) were better discriminated both by EEG and behavior than the taste pairs in the present study. Thus, by increasing participants’ average ability to discriminate a taste pair, the corresponding brain responses are easier to discriminate in terms of both accurateness and duration of the decoding.

We achieved a more fine‐grained analysis than Crouzet et al. (2015) by also considering the *individual* discriminatory ability of each participant. *Within* a taste pair, we separated participants according to their perceptual discriminatory ability, and interestingly, found a relation between behavior and EEG: participants who more consistently discriminated a taste pair perceptually also generally had brain responses that were more discriminable. We therefore provide the first proof of a functional relation between brain responses and perception of tastes on an individual level.

Yet, further studies are necessary to get a more complete understanding of subliminal taste processing. For example, studies on various subliminal taste qualities, intensities, and valences, in addition to the influence of other senses, such as somatosensation. The present study limited itself to self‐reported normal tasters, a selection criterion that could advantageously be confirmed by standardized taste tests and extended to address subliminal taste processing of subjects with taste disorders.

## AUTHOR CONTRIBUTIONS

*Conceptualization*: CA, MK, OL, TK, UK, SM, PK; *Methodology*: CA, MK, RA, SM, PK; *Software*: RA; *Investigation*: CA, MK, SM; *Formal Analysis*: CA, RA, PK; *Data Curation*: CA, MK; *Writing – Original Draft*: CA, RA, PK; *Writing – Review & Editing*: CA, MK, RA, OL, TK, UK, SM, PK; *Visualization*: CA, PK; *Supervision*: CA, OL, TK, UK, SM, PK; *Project Administration*: CA, MK, RA; *Funding Acquisition*: SM.

## CONFLICT OF INTEREST

The authors declare no competing interests.

## Supporting information

Transparent Science Questionnaire for Authors
